# Uncommon Metastasizing Site of Adrenocortical Carcinoma

**DOI:** 10.7759/cureus.15267

**Published:** 2021-05-27

**Authors:** Tarun Kumar, Jitendra S Nigam, Shambhwi Sharma, Madhu Kumari, Jagjit Pandey

**Affiliations:** 1 Pathology/Lab Medicine, All India Institute of Medical Sciences, Patna, IND; 2 Pathology/Laboratory Medicine, All India Institute of Medical Sciences, Patna, IND; 3 Surgical Oncology, All India Institute of Medical Sciences, Patna, IND

**Keywords:** endocrine, cytology, adrenal gland, bone, metastasis

## Abstract

Adrenocortical carcinoma (ACC) is a very rare tumor having a poor prognosis. Approximately 25% of these cases may present with metastases at the time of diagnosis. Common sites of metastasis are lung, liver, and lymph nodes. The bone metastases in ACC are less frequent. We report a case of a 35-year-old male presented with right parotid region swelling, rendered with a diagnosis of ACC metastasizing to the mandible ramus, which is an uncommon site.

## Introduction

Adrenocortical carcinoma (ACC) is an uncommon, aggressive malignant tumor with an incidence of 0.5-2.0 cases per million inhabitants per year [1-3]. ACC has a bimodal age distribution, with small peaks in the first decades and a larger peak in the fourth to fifth decades of life [2,3]. Patients with functional ACC may present with excess steroid hormone production resulting in Cushing syndrome with or without virilization [2,3], while non-functional ACC patients may present with either symptoms related to tumor mass, such as abdominal discomfort, nausea, vomiting, and back pain, or discovered incidentally during radiological procedures [2,3]. About one-fourth of ACC can present with metastases at the time of diagnosis [3]. The lung, liver, and lymph node are the common sites of metastasis [1-3]. Bone metastases (usually at pelvic bone and spinal bone) are infrequent and may present with fractures, spinal cord compression, or hypercalcemia [1-3]. We report a case of ACC with mandible bone metastasis (BM), an uncommon site.

## Case presentation

**Figure 1:**
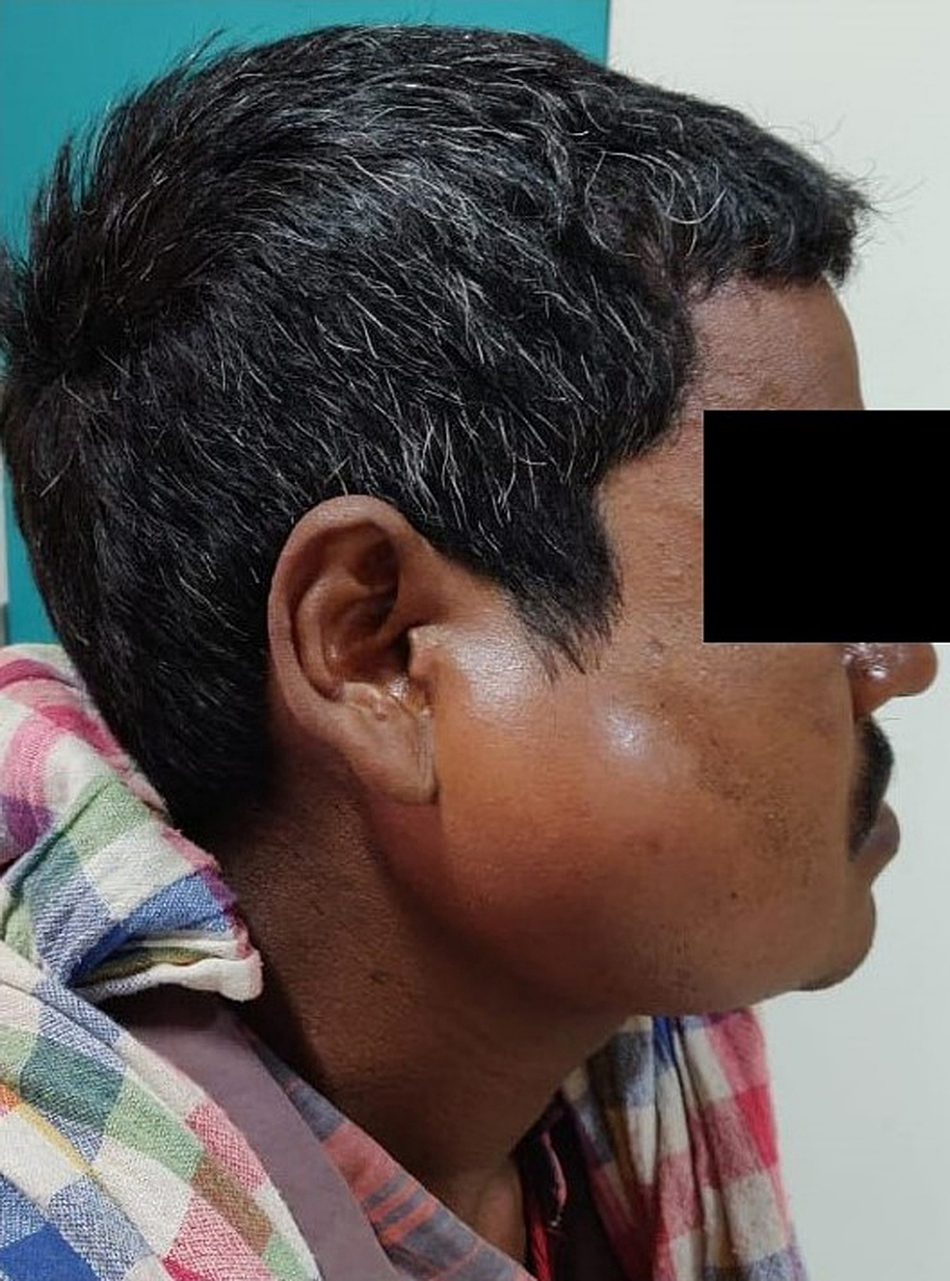
Clinical Image An ill-defined swelling over the right parotid region.

A 35-year-old male presented to the surgical oncology outpatient department with complaints of painless progressive ill-defined swelling measuring 7x6 cm over the right parotid region for three months. The swelling was firm to hard in consistency and fixed to the underlying structure (Figure 1). 

**Figure 2:**
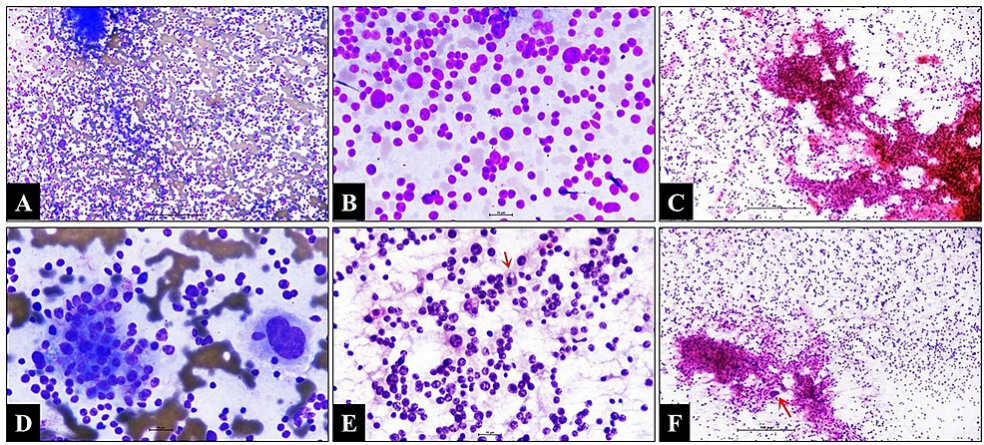
Cytology A,  B: Discohesive, round-to-polygonal tumor cells showing moderate nuclear pleomorphism (Giemsa; x100 and x400). C: Fibrovascular core traversing the tumor fragment (Papanicolaou; x100). D: The loose cohesive cluster of tumor cells with vacuolated cytoplasm and bizarre cells (Giemsa; x400). E: Mitotic figure (arrow) (Papanicolaou; x400). F: Tumor fragment with tumor giant cell (arrow) (Papanicolaou; x100).

The patient revealed a history of left radical nephrectomy six months back for renal cell carcinoma. No clinical manifestations of increased hormone secretion were noted. The fine-needle aspiration cytology showed mainly discohesive tumor cells displaying mild-to-moderate pleomorphic round-to-polygonal hyperchromatic nuclei. Few cells had irregularly clumped chromatin and prominent nucleoli. The cytoplasm was scant to moderate in amount. Occasional papillary fragments, multinucleated giant cells, bizarre cells, and mitosis were also noted (Figure 2). 

**Figure 3:**
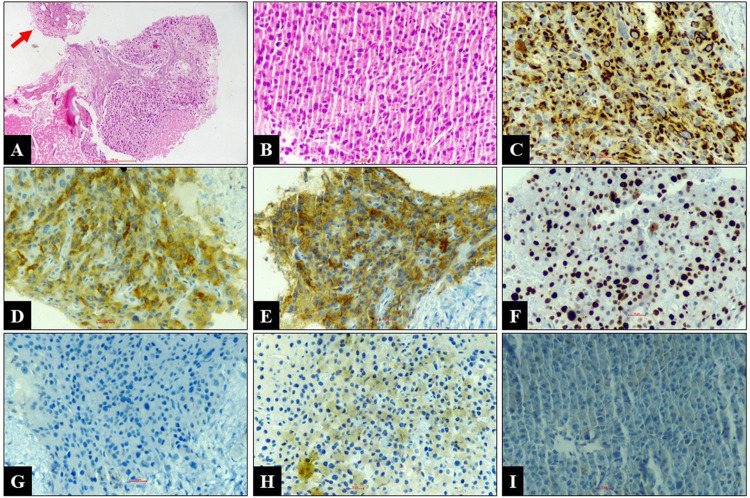
Histology and immunohistochemistry A: Tumor infiltrating the bone with areas of necrosis. Normal salivary gland acini also noted (arrow) (H&E; x100). B: Tumor cells are arranged in diffuse sheets showing moderate nuclear pleomorphism along with few mitotic figures (H&E; x400). C: Pan-cytokeratin cytoplasmic positive in tumor cells (x400). D: Inhibin cytoplasmic positive in tumor cells (x400). E: Synaptophysin cytoplasmic positive in tumor cells (x400). F: Ki-67 (approximately 60%) nuclear positive in tumor cells (x400). G-I: Chromogranin, PAX8, and CD117 negative in tumor cells (x400). H&E, hematoxylin and eosin.

Few of the tumor cells showed vacuolated cytoplasm. No dyskeratotic squamous cells, extracellular mucin, mucinophages, osteoid, or chondroid matrix were identified. Based on large polygonal cells, hyperchromatic nuclei, a moderate amount of cytoplasm, and the history of nephrectomy, the cytological differential diagnoses considered were metastatic renal cell carcinoma-chromophobe type, metastatic ACC, and pheochromocytoma. The true cut biopsy reveals a tumor arranged in the diffuse sheet. The cells displayed mild-to-moderate nuclear pleomorphism, hyperchromatic nuclei, and inconspicuous-to-prominent nucleoli. The cells had a moderate amount of eosinophilic cytoplasm. The attached bony fragments were also infiltrated by similar tumor cells with a large area of necrosis. An occasional salivary gland acinus was also noted. The tumor cells showed immunoreactivity for pan-cytokeratin, synaptophysin, and inhibin. They were negative for S100, chromogranin, CK7, CK20, PAX8, CD117, p40, p63, TTF1, CDX2, CD45, and vimentin. The Ki-67 labeling index was approximately 60%. Histomorphology and immunohistochemistry features were consistent with metastatic ACC (Figure 3). 

Since the patient's condition rapidly deteriorated, he left the institute against medical advice and was lost to follow-up.

## Discussion

ACC is an uncommon malignant epithelial tumor of adrenal cortical cells with female predilection and commonly arises in the left adrenal gland [1,2,4]. The ACC can be functional with clinical manifestations of excess hormone secretion, and hypercortisolism is the most common presentation in these cases [4-6]. However, non-functional ACC is presented with non-specific symptoms and affects males more than females [6]. Approximately 25%-35% of patients present with metastatic disease at the time of presentation [2,6]. Cytological features of primary ACC include cellular smear with cells arranged in loosely cohesive sheets, cords, or dispersed [7,8]. Cells are polygonal pleomorphic with eccentric hyperchromatic nuclei and prominent nucleoli [7,8]. The cytoplasm is eosinophilic granular to granular [7,8]. The background is hemorrhagic or milky along with stripped nuclei [7,8]. Occasional vacuolated cytoplasm, multinucleated tumor giant cells, mitotic figures, and traversing fibrovascular tissue fragments are also reported [7,8]. The lung, liver, and regional lymph nodes are the common metastatic sites while bone involvement mainly in the pelvis is infrequent [1-5]. Patients with BM may present with bone pain, pathological fractures, hypercalcemia, and spinal cord and nerve root compressions [1,6]. In the present case, the patient was a 35-year old who presented with ill-defined swelling over the right mandibular region with a history of left radical nephrectomy without any clinical symptoms of increased hormone secretion. According to the European network for the study of the adrenal tumors staging system, BM in ACC is considered stage IV [1,4,9]. The conventional, oncocytic, myxoid, and sarcomatoid are the histological variants [4,9]. They show immunopositivity for SF1, inhibin alpha, melan-A, synaptophysin, and calretinin [4,9]. In the present case, tumor cells were immunopositive for pan-cytokeratin, synaptophysin, and inhibin. Rachapalli et al. also report a case of ACC with metastasis to the mandible [10]. Surgical resection is the cornerstone of the management in ACC. If both the metastatic and primary lesions are to be removed entirely, then surgical resection may be considered at the metastatic site [6]. The chemotherapy drug, such as mitotane, plays a role in incompletely removed or metastatic ACC by impeding the steroid biosynthesis and damaging adrenocortical cells [5,11]. Mitotane can be used either in combination with other cytotoxic drugs or alone, with response rates ranging from 12% to 54% [6,11]. The tyrosine kinase inhibitors and rapamycin signaling components may also show some agreement in the management of ACC; however, their role is still uncertain [3,11]. Shuzhong et al. achieve the therapeutic effect of anti-PDL-1 in clinical and radiological manifestations in ACC with spine metastasis [11]. Radiotherapy may be used as palliative treatment in bone and brain metastases, as well as in postoperative recurrences. For patients who are poor for surgery but have metastatic lesions <5 cm, radiofrequency ablation is another option [6].

The five-year survival rate of patients with ACC is <50%. Clinical stage, grade, resection status, patient age, tumor capsule status, Ki-67 index, and symptoms related to the tumor or hormone excess are the important prognostic factors [4,9]. Guillaume et al. stated that a combination of the number of tumoral organs at the time of the first metastasis and the mitotic rate could help to predict the disease outcome [12].

Patients with <2 tumoral organs with metastases and <20 mitoses/high-power field (HPF) in the primary tumor may have a relatively better five-year survival than patients with metastatic diseases with increased tumor burden [12]. Other predictive factors include hepatic and bone metastases, number of metastatic lesions, number of tumoral organs involved at the first metastasis, a high mitotic rate (>20/50 HPF), and atypical mitoses in the primary tumor [12]. In the index case, the patient's condition deteriorated rapidly and left the institute without further management.

## Conclusions

ACC is an exceedingly rare tumor with a poor prognosis and may present as only BM without involving other common metastasis sites with or without hormone-associated symptoms. ACC may be considered as a differential diagnosis in cases of mandible BM of unknown origin and these cases may present with the painless progressive ill-defined, firm-to-hard swelling. The mandible ramus is an uncommon site for ACC metastasis as reported in this case.
